# Interactions between HIV proteins and host restriction factors: implications for potential therapeutic intervention in HIV infection

**DOI:** 10.3389/fimmu.2024.1390650

**Published:** 2024-08-16

**Authors:** Farooq Rashid, Silvere D. Zaongo, Hifza Iqbal, Vijay Harypursat, Fangzhou Song, Yaokai Chen

**Affiliations:** ^1^ Department of Infectious Diseases, Chongqing Public Health Medical Center, Chongqing, China; ^2^ School of science, University of Management and Technology, Lahore, Pakistan; ^3^ Basic Medicine College, Chongqing Medical University, Chongqing, China

**Keywords:** HIV, HIV proteins, host restriction factors, HIV infection, therapeutics

## Abstract

Different host proteins target different HIV proteins and antagonize their functions, depending on the stage of the HIV life cycle and the stage of infection. Concurrently, HIV proteins also target and antagonize various different host proteins to facilitate HIV replication within host cells. The preceding quite specific area of knowledge in HIV pathogenesis, however, remains insufficiently understood. We therefore propose, in this review article, to examine and discuss the HIV proteins that counteract those host restriction proteins which results directly in increased infectivity of HIV. We elaborate on HIV proteins that antagonize host cellular proteins to promote HIV replication, and thus HIV infection. We examine the functions and mechanisms via which Nef, Vif, Vpu, Env, Vpr, and Vpx counteract host proteins such as Ser5, PSGL-1, IFITMS, A3G, tetherin, GBP5, SAMHD1, STING, HUSH, REAF, and TET2 to increase HIV infectivity. Nef antagonizes three host proteins, viz., Ser5, PSGL1, and IFITIMs, while Vpx also antagonizes three host restriction factors, viz., SAMHD1, STING, and HUSH complex; therefore, these proteins may be potential candidates for therapeutic intervention in HIV infection. Tetherin is targeted by Vpu and Env, PSGL1 is targeted by Nef and Vpu, while Ser5 is targeted by Nef and Env proteins. Finally, conclusive remarks and future perspectives are also presented.

## Introduction

Human immunodeficiency virus (HIV) is a single-stranded RNA virus, and its RNA, upon entry into the host cell, is reverse transcribed into DNA, which subsequently integrates into host DNA (1). Helper T-lymphocytes and monocytes are the primary targets of HIV-1, and from the point of the attachment of HIV to the host cell to the production of viable HIV virions, several host restriction factors also participate in the life cycle of HIV (2, 3).

Within infected cells, a number of host proteins are expressed in response to HIV infection, which inhibit HIV replication at various stages of the life cycle of HIV. Since these host proteins restrict HIV replication within cells, the host proteins are referred to as restriction factors, and participate in host defenses against all viral infections, including HIV infection (4, 5). Host restriction factors inhibit infections by recognizing and interfering with specific steps in the life cycle of the virus responsible for the infection. Certain specific processes such as the expression of host restriction proteins within different cell types, the rapid action of restriction proteins, and their inducibility by interferons (IFNs) may enable host restriction proteins to acquire an enhanced potency to inhibit viral infections, (6).

Up until now, several restriction factors that inhibit HIV-1 infection at various stages and which target different proteins of HIV have been described. Some of the restriction factors may target particular HIV proteins. A few of the host restriction factors include Serine incorporate 5 (Ser5) (7), P-selectin glycoprotein ligand 1 (PSGL-1) (8, 9), interferon-induced transmembrane (IFITM) proteins (10), Apolipoprotein B mRNA editing enzyme, catalytic polypeptide-like 3G (APOBEC3G) or A3G (11), Tetherin (12, 13), Guanylate-Binding protein 2 and 5 (GBP2/5) (14), RNA-associated early-stage antiviral factor (REAF) (15), member of ten-eleven translocation 2 (TET2) (16), Sterile Alpha Motif and Histidine Aspartate domain containing protein 1 (SAMHD1), stimulator of IFN genes (STING), and human silencing hub (HUSH) restriction to promote HIV-2 replication (17–19).

HIV has evolved and has adapted to the human host in a manner that allows it to counteract certain host restriction factors in order to increase the success of its infection. Several HIV proteins, such as Nef, Virion infectivity factor (Vif), viral protein U (Vpu), Envelop protein (Env), viral protein R (Vpr), and viral protein X (Vpx) are known to counteract the effects of host restriction factors via different mechanisms. Indeed, Nef interacts with and downregulates the expression of Ser5, PSGL1, IFITM proteins (7, 9, 20), while Vif degrades APOBEC3G (7, 9, 20, 21), Vpu antagonizes tetherin, PSGL1, and GBP5 (8, 22–25), Env restricts tetherin and Ser5 (26–28), Vpr antagonizes REAF and TET2; and Vpx overcomes SAMHD1, STING, and HUSH restriction to promote HIV-2 replication (17–19). The restriction of these host factors by HIV proteins helps HIV to propagate and sustain a productive infection within the human host.

In order to counteract HIV/AIDS, it is fundamentally important to understand how the human host cellular machinery is exploited by HIV for its own benefit. HIV-1 is known to interact with host proteins, and dissection and elucidation of the underlying mechanisms integral to these interactions would be of significant importance for the development of novel strategies to control HIV. In the present review article, we discuss how HIV proteins are able to counteract host restriction factors in order to establish a successful infection, and offer perspectives related to the preceding discussion.

## Nef downregulates Ser5/3, cell surface PSGL-1, and IFITMs to increase HIV-1 infectivity

HIV-1 Nef encodes 200 to 215 amino acid residues, is the most abundantly transcribed gene during the early stages of HIV infection, and is important for viral replication (29–31). The Nef protein contains globular core residues of 58-149 and 180-206 amino acids, an N-terminal anchor domain (amino acids 1-58), and an internal flexible loop (amino acids 149-179) (29). The C-terminal flexible loop contains a dileucine motif [(D/) ExxxLL,160–165] which binds endocytic adaptor protein (AP) complexes (32, 33), and two diacidic motifs, i.e., (E154, 155) and (E/DD174, 175), which interact with the beta subunit of the coatomer protein (β-COP) (34) and the vacuolar ATPase catalytic subunit (35), respectively. Nef is a multifunctional protein that is involved in several activities within the cell, including the downregulation of T-cell receptors, of MHC-1, and of CD4+ T-cells, and thus tends to increase HIV infection (36). Other functions of Nef include modulation of the activation state of macrophages and T-cells, and perturbation of the actin cytoskeleton (7, 37). A further function of Nef was described in 1994, i.e., its ability to increase virion infectivity, and this function is conserved among primate lentiviruses (38, 39). Viruses lacking Nef have been found to have reduced infectivity, thus underlining the role of Nef during viral infections (39). Nef is one of the earliest HIV genes, resulting in the modification to that leads to viral replication. Nef is detectable in the HIV-1 infected patients serum, even when plasma RNA levels of HIV-1 are not detectable (40).

**Figure 1:**
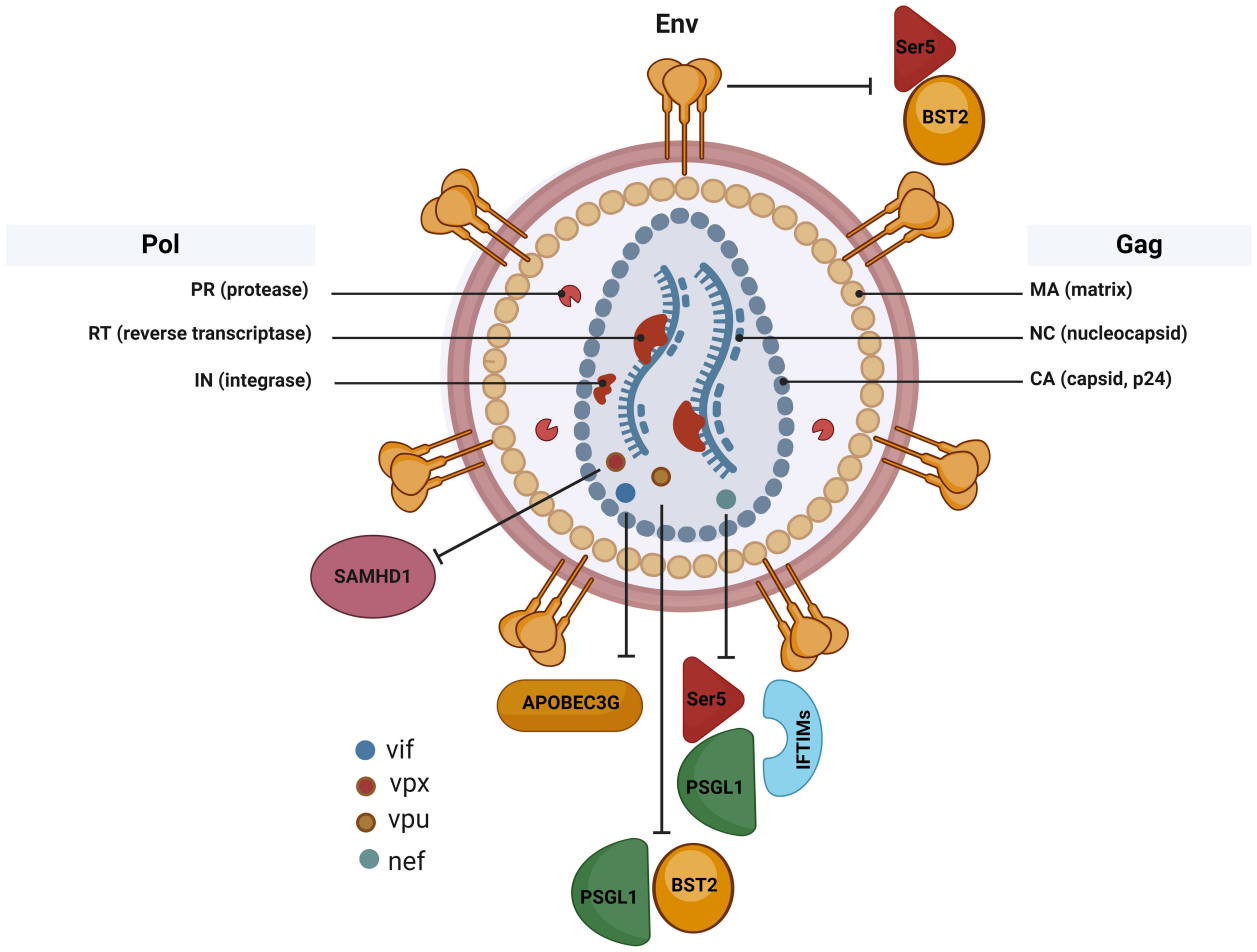
Counteraction of host restriction factors by HIV proteins. Nef downregulates Ser5; Nef binds and directly downregulates Ser5 at steady-state levels via the lysosome/endosome system Nef internalizes Ser5 via receptor-mediated endocytosis. In the absence of Nef, Ser5 cannot be internalized cell surface PSGL-1; Nef downregulates surface PSGL-1 to enable HIV to partially escape PSGL-1-mediated restriction and IFITMs; Nef overcome the restriction imposed by IFITM-mediated inhibition on both HIV-1 and HIV-2. Nef increases HIV-1 production during IFITM1 and IFITM2 expression to increase HIV-1 infectivity, Vif increases HIV-1 infectivity by degrading APOBEC3G; Vif binds and counteract human APOBEC3G leading to its poly-ubiquitination and proteasomal degradation and thus promotes viral replication Vpu antagonizes tetherin by physically interacting with it; Vpu binds PSGL1 and induce its ubiquitination and degradation to increase HIV-1 infection. Envelop (Env) restricts tetherin; Env interacts with tetherin and sequester it and inhibit its trafficking to plasma membrane and this redistribution correlates with tetherin antagonism and thus viral infectivity is increased; Env antagonizes Ser5 without inhibiting Ser5 virion incorporation to increase HIV infection. Vpx overcomes SAMHD1 restriction to promote HIV-2 replication. Vpx physically interact with SAMHD1, recruiting the Cullin-4 E3 ubiquitin ligase complex, and destines SAMHD1 for poly-ubiquitination and proteasomal degradation to increase HIV-2 replication.

Transcriptomic analysis of low and high Nef-responsive cells have identified Ser5 as a protein that may putatively be responsible for regulation of the HIV-1 infectivity which correlates with Nef responsiveness. Nef increases the infectivity of viral particles by downregulating Ser5 (**Table 1**, **Figure 1**) (7). The Ser protein family has five protein members (Ser1 to Ser5). Serinc proteins are membrane proteins that flip lipids, and eventually leading into loss of membrane asymmetry which is related with the loss of infectivity (48). Ser3 and Ser5 are found to increase the signaling of IFN-I and NF-κB (49). Ser5 interacts with mitochondrial membrane antiviral signaling protein (MAVS) and TRAF6, the important members of innate immune signaling. Serinc proteins are not induced by IFN, and are therefore classified as non-classical host restriction factors. Both ser3 and ser5 boost the innate immune signaling, and therefore increases the production of IFN-I and pro-inflammatory cytokines. These events are found not only in the perspective of HIV-1, but also for zika virus (ZV) and vesicular stomatitis virus (VSV) (49). Ser5 has been established as a host restriction factor that inhibits HIV-1 replication. Among the various isoforms of ser5, the longest isoform (Ser5-001) is stably expressed. Ser5-001 is the predominant antiviral isoform that is incorporated into HIV-1 particles, and inhibits HIV replication at early stages of the HIV life cycle (50, 51). However, it has been observed that Nef binds and directly downregulates Ser5 at steady-state levels via the lysosome/endosome system, to effectively counteract the expected inhibition of HIV by Ser5.Cell surface levels of serinc proteins are decreased by the Nef expression (52) and specifically, Nef internalizes Ser5 via receptor-mediated endocytosis. In the absence of Nef, Ser5 cannot be internalized. Moreover, the polymorphisms that occur in Nef impairs its ability to internalize Ser5; thus, mutations leading to variations in Nef protein expression may likely contribute to observed variations in viral pathogenesis (41). Linked genotype-phenotype dataset analysis revealed that 18 polymorphism occurring naturally on 14 codons were associated with differential Ser5 down regulation (41).

**Table 1:** HIV proteins and their antagonized host restriction factors.

Viral protein	Counteracting host restriction factor	Mechanism	Reference
Nef	Ser5 PSGL-1 IFITMS	Nef binds Ser5, internalizes, and downregulates Ser5 expression	(41)
		Nef downregulates PSGL-1 and may redirect PSGL-1 to intracellular compartments	(8, 9)
		Nef downregulates IFITMs by altering their subcellular distribution	(20, 40)
Vif	A3G	Vif binds with A3G, and induces the poly-ubiquitination and proteasomal degradation of A3G	(11, 21)
Vpu	Tetherin PSGL-1	Vpu interacts with tetherin, and induces the ubiquitination and degradation of tetherin	(22, 25, 42, 43)
		Vpu interacts with PSGL-1 and triggers the ubiquitination and degradation of PSGL-1 through SCF^β-TrCP2^	(8, 9)
	GBP5	Vpu counteracts GBP5 indirectly	(44)
Env	Tetherin Ser5	Env interacts with tetherin and confines tetherin to the *trans-*Golgi network, and thus sequesters it from virus assembly sites on the plasma membrane	(26)
		Unknown mechanism	(27, 28)
Vpr	REAF TET2	Vpr interacts with and degrades REAF in primary macrophages	(45)
		Vpr degrades TET2 via VprBP-DDB1-CUL4-ROC1 E3 ligase	(16)
Vpx	SAMHD1 STING HUSH	Vpx interacts with SAMHD1, recruits Cullin-4 E3 ubiquitin ligase complex, destines SAMHD1 for poly -ubiquitination and proteasomal degradation	(17–19)
		Vpx interacts with STING and inhibits NF-κB activation	(46)
		Vpx interacts with HUSH complex, and recruits DCAF1 ubiquitin ligase adopter to degrade HUSH	(47)

Future studies in this regard may enhance our understanding of the Nef-Ser5 interface at molecular levels, and this may represent an attractive option for the potential revelation of new antiviral therapeutics. There is more than 17% of amino acid similarity among the five human Ser family members; therefore, in the study mentioned previously (7), the other Ser family members were explored because of their involvement in anti-HIV-1 activities. Overexpression of Ser3 results in a threefold decreased production of Nef-defective HIV-1, suggesting the role that Ser3 may play in HIV-1 infectivity inhibition. The infectivity inhibition attributable to Ser3, however, occurs to a lesser degree compared to Ser5, which is also counteracted by Nef (7).

As described above, the key role of Nef is in increasing virion infectivity via downregulation of Ser5. However, one research group have sought to determine whether Nef contributes significantly to HIV-1 subtype C disease progression (53). Using a flow cytometry-based assay, the potential of 106 Nef clones (isolated from patients in early infection) to downregulate Ser5 in a CEM (acute lymphoblastic leukemia T-lymphoblast) -derived CD4+ T-cell line was evaluated in order to test their hypothesis (41).

Codon-by-codon analysis revealed that amino acids 10I, 11V, 38D, 51T, 65D, 101V, 188H, and 191H were responsible for an increase in Ser5 downregulation, whereas amino acids 10K, 38E, 65E, 135F, 173T, 176T, and 191R were associated with decreased downregulation. Further experiments with site directed mutagenesis delineated that Ser5 down regulation was associated with 173T mutation, however, 10K, 135F, and 176T mutations were associated with non-significant reductions (53). The Nef mediated Ser5 sequence determinants were determined which showed that 15 amino acid polymorphisms at 11 different codons were associated with Ser5 down regulation activities. The amino acid variations at codons 10, 11, 38, and 173 were found to be associated with more than 30% Ser5 down regulation (53). Thus, it was inferred that Ser5 downregulation contributes to overall Nef activity and function. Furthermore, the potential genetic determinants of Nef activity and function may be an important consideration for future therapeutics and vaccine development (53).

Liu et al., infected CD4+ T-cells with HIV-1 in an attempt to isolate new anti-HIV-1 factors. For this purpose they used isobaric tag-based quantitative mass spectrometry (MS) technology, and quantified approximately 14000 proteins (8). For comparison, RNA sequencing was performed, and it was observed that expression of a wide range of proteins was altered in response to HIV-1 infection. Among these proteins, PSGL-1 was focused on for further study. PSGL-1 is a 120-kDa glycoprotein that is expressed on the surface of myeloid and lymphoid cells. PSGL-1 inhibits HIV-1 infectivity by blocking HIV virion attachment to target cells. PSGL-1 is increased during inflammation to regulate leukocyte tethering and thus to promote cell migration into flamed tissues. During T cell differentiation, the IFN-γ and IL-12 promote PSGL-1 expression, indicating that PSGL-1 could be an IFN-γ-regulated factor (9). Furthermore, to determine the domain responsible for its anti-viral activity, the deletion mutagenesis of this protein domain was performed. It was revealed that rigid and elongated extracellular N-terminal domain of PSGL-1 is indispensable for its anti-HIV-1 activity. (9). Experimental results have indicated that levels of PSGL1 proteins (but not PSGL1 mRNA levels) were downregulated after HIV-1 infection of cells (8). Fu et al., also determined that PSGL-1 blocks HIV-1 attachment to cells and thus decreases HIV infectivity (9). Nef is a broad-spectrum regulator of cell-surface receptors (54–56). Thus, the role played by Nef in HIV-1-mediated downregulation of PSGL-1 was explored (9). Fu et al., determined that HIV-1 Nef downregulates surface PSGL-1 in a dose-dependent manner to enable HIV to partially escape PSGL-1-mediated restriction (**Table 1**, **Figure 1**); however, Nef is unable to decrease intracellular PSGL-1 levels, and may redirect intracellular PSGL-1 to intracellular compartments (9).

Interferon-induced transmembrane (IFITM) proteins inhibit cellular entry of various viruses including dengue virus, influenza A H1N1 virus, and West Nile virus (57). Furthermore, it was observed that IFITM may also inhibit HIV-1 infection by interfering with HIV entry, as IFITM proteins are membrane-associated proteins (10). This inhibition of HIV-1 entry by IFITM proteins is regulated by a virus co-receptor. Notably, CCR5-tropic strains are more sensitive to IFITM1 while CXCR4-tropic strains are more sensitive to IFITM2 and IFITM3 (58–60). Apart from its role in inhibiting the cellular entry of HIV-1, IFITM proteins also inhibit viral replication, as knockdown of IFITM1 has been seen to increase HIV-1 titers (20). Indeed, IFITM expression correlates with reduced HIV-1 Gag levels, indicating a reduction in viral protein synthesis that is independent of inhibition of virus entry (20). However, in the preceding study, the authors also determined that Nef may overcome the restriction imposed by IFITM-mediated inhibition on both HIV-1 and HIV-2 (**Table 1**, **Figure 1**). They observed that Nef increases HIV-1 production by approximately 4-fold during IFITM1 and IFITM2 expression. Moreover, the cells producing Nef-deleted virus in the presence of IFITM expression produced significantly lower levels of HIV-1 Gag (p55). The restriction of IFITM proteins may not be via degradation, as exogenous IFITM levels are not reduced by HIV-1 Nef. Although it is not clear how Nef counters IFITMs, a possible explanation could be re-trafficking, wherein Nef relocates IFITMs away from their sites of action on translation.

One recent investigation has outlined the function of Nef in extracellular vesicles (EVs). The study performed a proteomic analysis of T-cell-derived EVs to characterize Nef-induced changes in proteomic content (40). Among the various downregulated proteins, IFITIM1/2/3 were also significantly downregulated. The levels, in EVs, of all three IFITMs were depleted by Nef, suggesting that Nef is a modulator of EV proteomic content, and antagonizes IFITMs by altering their subcellular distribution (40).

## Vif increases HIV-1 infectivity by degrading A3G

Virion infectivity factor (Vif) contains 190-240 amino acid residues, and is encoded by all lentiviruses, with the exception of the equine infectious anemia virus (61). The Simian Immunodeficiency Virus, SIVmac239 clone [which is deficient in Vif (*delta*vif)], does not cause disease; however, SIVmac239 *delta*vif clone causes HIV-1 to replicate at lower titers (62). *Delta*Vif HIV-1 clone does not replicate in non-permissive cell lines; however, it does replicate in permissive cell lines. Non-permissiveness, in this context, is due to the expression of an inhibitory factor, APOBEC3G, also called A3G. Thus, due to the expression of APOBEC3G, some cell lines become non-permissive (63).

A3G, discovered in 2002, is a member of the family of RNA-editing enzymes which induces hypermutations in HIV-1 DNA by cytidine deaminase activity, and eventually results in the degradation of viral DNA (11, 64). However, if the hypermutated viral DNA integrates into the host genome, this DNA is unable to code functional viral proteins (65, 66). HIV-1 depends on Vif to counteract the antiviral effect of A3G. However, it has been observed that Vif interacts only with human A3G, and not with mouse, African green monkey (AGM), and rhesus macaque A3G (67). Consequently, A3G from these animals are potent inhibitors of viral replication, as A3G from these animals are unable to form complexes with Vif (67). HIV-1 Vif binds with A3G and recruits A3G to the Cullin5-based E3 ubiquitin ligase complex (**Table 1**, **Figure 1**). This recruitment results in poly-ubiquitination and proteasomal degradation of A3G, and promotes viral replication (11, 21). The central domain of this complex is Vif heterodimer with core binding factor subunit β (CBF-β), which is transcription factor, stabilizing Vif. Since endogenous A3G protein adds to HIV-1 restriction in THP-1 cells, HIV-1 virions lacking Vif have 50% lower infectious rate compared to wild type HIV-1 (68). Recently, HIV-1 Vif mutants were used to determine the effects of other A3 proteins on HIV-1 infectivity. For this purpose, A3F, A3F/A3G, and A3A to A3G null THP-1 cells were developed. The HIV-1 lacking Vif infectivity was robustly inhibited in A3F null THP-1 cells and less inhibited in A3F/A3G null THP-1 cells compared to wild type HIV-1. Moreover, the infectivity of HIV-1 lacking Vif was comparable to wild type HIV-1 in A3A to A3G null THP-1 cells. Therefore, it could be inferred that HIV1 Vif primarily target A3 proteins during infectious virus production from THP-1 cells (68).

Different host proteins are known to regulate Vif activity and thus the antiviral behavior of A3G in different ways to affect HIV-1 pathogenesis. For example; On the one hand, mouse double minute 2 (MDM2) homolog, an E3 ligase, increases A3G levels by binding Vif by promoting its ubiquitination, followed by its proteasomal degradation (69). While, core binding factor β (CBFβ), on the other hand, stabilizes Vif and therefore inhibits the antiviral effects of A3G (70). Similarly, apoptosis signal-regulating kinase-1 disrupts the interaction between A3G and Vif to facilitate A3G antiviral activity (71). Vif has a putative AKT phosphorylation motif (RMRINT), and is phosphorylated by AKT at threonine 20, which enhances its stability and in turn its viral infectivity by degrading A3G. Vif becomes destabilized when threonine 20 is replaced by alanine. Furthermore, inhibition of AKT downregulates or decreases the stability of Vif, which increases antiviral activity by restoring A3G levels (72). Therefore, the Vif activity is regulated by different host proteins and thus this regulation affects the antiviral activity of A3G and hence HIV-1 infectivity.

## Vpu antagonizes tetherin, PSGL-1, and GBP5 to increase HIV-1 infection

Vpu contains 81 amino acid residues, and is a multimeric integral membrane phosphoprotein (13). The Vpu gene is present in HIV-1, and is absent in HIV-2, rhesus macaque SIV (SIVmac), and sooty mangabey SIV (SIVsmm) (73–75). Vpu is mainly present in plasma membranes, Trans-Golgi network (TGN) and endoplasmic reticulum (ER) (13). Vpu has several functions, that included;, it increases the degradation of CD4 protein through the ubiquitin-proteasomal pathway, it enhances the release of progeny virions from infected cells by counteracting the host protein, tetherin (76), it regulate MHC II presentation (77), modulates the transportation of host proteins from ER to Golgi (78), increases the stabilization of p53 (79), enhances the de-granulation of natural killer cells (80), induction of apoptosis (81), and the lipid antigen presentation by down regulating CD1d (82) However, the function of Vpu in virus release differs in different cell types. The Vpu-induced release of HIV-1 from HIV-infected HeLa cells and T cells is increased, but is not affected in COS, CV-1, HEK293T, and Vero cells (13, 83).

Tetherin (BST2 or CD317), is a host restriction factor that inhibits the release of virions from HIV-infected HeLa cells, but not from infected HEK293T cells (12, 13). The cytoplasmic tail (which contains 21 amino acid residues), the TM domain (which contains three cysteine residues that mediate tetherin dimerization) (84), an extended coiled-coil (85), and a C-terminal glycophosphatidylinositol anchor (86) are the key structural features contributing to the mode of action of tetherin (84). Moreover, overexpression of tetherin in HEK293T cells inhibits the secretion of virus particles in the absence of Vpu, while knockdown of tetherin in HeLa cells results in the release of Vpu-defective virion particles (12).

Vpu and tetherin physically interact with each other and as a result tetherin is counteracted (**Table 1**, **Figure 1**) (22). In order to find out the motif responsible for the interaction, mutations were generated i.e. S52A and a double mutation-S52A and S56A (2S/A), that are crucial for β-TrCP recruitment, followed by analyses to whether these mutated Vpu constructs could deplete tetherin in 293T cell. Interestingly, both of these constructs were unable to decrease the tetherin levels. However, further analysis showed that Vpu motif-β-TrCP recruitment motif was indispensable for to counteract tetherin. Furthermore, β-TrCP was found to be co-immunoprecipitated with tetherin, followed by the proteosomal degradation of tetherin in a β-TrCP dependent manner (22).

Furthermore, the amino acids were determined responsible for interaction between Vpu and tetherin (43). The Ala14, Ala18, and Trp22 residues in the TMD of Vpu binds with the I34, L37, L41, and T45 residues in the TMD of tetherin, and mediates the downregulation of tetherin (42, 43). Vpu antagonizes tetherin via two sequential steps, i.e., 1. Cell surface down regulation of tetherin, which is regulated by clathrin-coated vesicles by a direct binding of AP2 with the Y6XV8 motif of tetherin (23, 87), 2. Restriction of the recycling of internalized tetherin to the cell membrane blocks the translocation of *de novo* tetherin (24, 88–90), and as a result the levels of tetherin are reduced. Vpu degrades tetherin via ubiquitination, and Vpu enhances its ubiquitination through lysine/serine and threonine residues present in the Vpu cytoplasmic tail (25). The HIV-1 VpuM group is a highly transmissible and pathogenic group among all HIV-1 groups, and the reason for this may be that the Vpu present in group M HIV-1 antagonizes tetherin to a much greater extent, and thus effectively evades the host immune system (13). However, HIV-1 group P Vpu does not antagonize tetherin cell surface expression, as the Vpu of group P HIV-1 does not contain the AxxxAxxxW motif, which is responsible for interaction with tetherin (42, 91). This may well be an explanation as to why HIV-1 group P has not as yet adapted as well to the human host as HIV-1 group M has (91).

Isobaric tag-based mass spectrometry was used to determine novel host HIV restriction factors in human CD4+ T-cells (8). During the preceding study, and in another study (9), PSGL-1 was identified as a novel HIV-1 restriction factor that is induced by IFN-γ, and blocks HIV-1 reverse transcription soon after the virus enters the cell (8). Infection of Jurkat cells with vesicular stomatitis virus G protein-pseudotyped HIV-1 lentiviral vector encoding green fluorescent protein (VSV-G-HIV-GFP) induces a decrease in PSGL-1 levels; therefore, different HIV proteins, i.e., p55 Gag, Vpr, Vif, Nef, and Vpu were analyzed to assess whether any one of these may be responsible for the downregulation of PSGL-1. Among these proteins, only Vpu was observed to be responsible for this action in Jurkat and 293T cells (8). HIV-1 infection in CD4+ T-cells also decreases endogenous PSGL1 levels significantly (8). Similarly, Vpu-deficient HIV (NL4-3delVpu) does not affect PSGL-1 levels (8). Moreover, co-immunoprecipitation experiments confirmed a direct interaction between Vpu and PSGL-1 (8). Vpu is known to hijack SCF^β-TrCP1/β-TrCP2^ E3 ligase (22); therefore, the possibility of Vpu degradation of PSGL-1 through E3 ligase was also tested. It was observed that, indeed, Vpu does induce PSGL-1 ubiquitination and degradation via SCF^β^-^TrCP2^, and furthermore, siRNA knockdown of β-TrCP2 reverses this Vpu-induced PSGL-1 degradation (8). Similarly, Vpu mutants (S52/56N or S52N/S56N) that are defective for β-TrCP1/2 binding do not promote PSGL1 ubiquitination and degradation (8). These results suggest that PSGL-1 is a potent HIV-1 host restriction factor that is antagonized by HIV-1 Vpu by its interaction with Vpu, and triggers the ubiquitination and degradation of PSGL-1 through SCF^β-TrCP2^.

Guanylate-Binding Protein-5 (GBP5) is a member of the IFN-inducible guanosine triphosphatase (GTPase) superfamily that plays a role in intrinsic immunity against bacteria, protozoa, and viruses. Guanylate-binding proteins (GBPs) hydrolyze guanosine triphosphate (GTP) to guanosine diphosphate (GDP) and guanosine monophosphate (GMP) (92). GBP5 is primarily expressed in cytosol and endosomal membranes. However, upon HIV-1 infection, GBP5 co-localizes with HIV-1 and reduces the production of infectious HIV-1 particles (93). According to analysis of the Genomic Utility for Association and Viral Analysis in HIV (GuavaH) database, higher levels of GBP5 are expressed in HIV-1 patients (94, 95). One study has suggested that HIV-1 Tat protein may increase GBP5 expression in human primary T-cells (96).

GBP affects the processing of HIV-1 Env in the Golgi apparatus by interfering with its N-linked oligosaccharide glycosylation, and this glycosylation is important for Env processing (44). This activity increases the incorporation of unprocessed immature gp160 into progeny virions, therefore impairing its trafficking to the cell surface and thus decreasing virion infectivity (44). However, there exists an indirect method of evading GBP5 inhibition which exploits naturally occurring mutations in the start codon of the HIV-1 Vpu gene in both macrophage (M)-tropic and brain derived HIV-1 strains. The HIV-1 Env protein is translated along with Vpu from a single bicistronic mRNA transcript (97). Thus, mutations in the Vpu start codon lead to enhanced expression of Env protein, countering GBP5 inhibition but consequently exposing the virus to being susceptible to restriction by tetherin, as Vpu is known to counteract the tetherin host restriction factor. These activities block the release of progeny virions and increase their accumulation on the host cell surface (44).

## Envelop protein restricts tetherin and Ser5 to increase HIV infection

Envelop protein (Env) plays an important role in the attachment of HIV to its host cell receptor (5). Env is glycosylated, plays a crucial role in the proper configurational folding of native protein, and facilitates host immune evasion, and thus, viral infection (98). HIV-2 Env glycoproteins have Vpu-like functions, which trigger the release of Vpu-defective HIV-2 virions from tetherin-positive cells (99). HIV-2 Rod envelop glycoprotein (HIV-2 Rod Env) counteracts tetherin-mediated restriction of Vpu-defective HIV-1 in a type-specific manner, but not HIV-1 or SIVmac1A11. In HIV-1 infection, Vpu is responsible for counteracting tetherin (26). Antagonism of tetherin by HIV-2 Env protein is due to extracellular determinants present in HIV-2 Env. Co-immunoprecipitation experiments conducted confirmed binding between tetherin and Env; however, tetherin is not degraded as a consequence of this bonding. Therefore, it could be inferred that HIV-2 Env may sequester tetherin. When over expression of HIV-2 Env, tetherin was distributed to perinuclear compartments and inhibiting its trafficking to plasma membrane and this redistribution correlates with tetherin antagonism (26).Env restricts tetherin to the *trans-*Golgi network, and therefore Env sequesters tetherin from virus assembly sites on the plasma membrane (26).

HIV-1 Env also restricts Ser5 (**Table 1**, **Figure 1**), via a mechanism that differs from Nef antagonism of Ser5 (28). The HIV-1 strains, AD8-1 and YU-2 (however not strain NL4-3), were found to be resistant to ectopic expression of Ser5, and interestingly, Nef protein from these strains was unable to inhibit the activity of Ser5 (28). The Env protein from some HIV-1 strains have been found to resist Ser5 inhibition (7, 27). The important functional regions in Env protein are the V1, V2 and V3 loops; however, the V3 loop was found to be a key player within Env protein that renders viral resistance to Ser5 (28). Furthermore, HIV-1 subtype A, C, and D Env proteins have been observed to be far more resistant to Ser5 inhibition compared to subtype B strains, suggesting a subtype-specific resistance of Env to Ser5 (28). However, Env proteins are unable to prevent the incorporation of Ser5 into virions (27, 28). These observations suggest that HIV-1 Env protein antagonizes Ser5 without inhibiting Ser5 virion incorporation. Furthermore, Ser5 disrupts the fusogenisity of Env glycoproteins. The confirmation of Env glycoprotein is altered upon Ser5 incorporation into viral membrane. Ser5 causes increased functional inactivation of Env with passage of time and inhibits HIV-1 fusion at a pre-hemifusion stage. Although it has been determined Env and Ser5 interact with each other (100), the experimental analysis revealed by (101) did not delineate any colocalization of these two proteins on HIV-1 pseudoviruses. It is therefore indispensable to resolve the issue whether Env interacts directly with ser5.

## Vpr interacts with and degrades REAF and TET2 to promote HIV-1 replication

Vpr is a 14kDa nonstructural protein of HIV-1 that is known for its function to trigger cell cycle arrest at the G2/M phase, and is required for viral replication (45, 102). Vpr triggers ubiquitin/proteasome dependent degradation of several host proteins, and in doing this activity Vpr increases HIV-1 gene expression and induces (G2/M) cell cycle arrest. Vpr causes the depletion of CCDC137/cPERP-B, a chromosomal periphery protein causing G2/M cell cycle arrest. On the contrary, the G2/M cell cycle arrest was not occurred in the presence of CCDC137 mutants, resistant to Vpr (103). A significant amount of Vpr is incorporated into virions and released from major capsid protein (CA) after cell entry. The timing of reverse transcription initiation and the release of Vpr from CA are identical; therefore, Vpr performs its function prior to initiation of the viral integration process (45).

A3G has been known to be encountered by Vif protein, another group (104) has reported that Vpr interacts with A3G and counteract its antiviral effect. An *in silico* method-PRISM (protein interactions by structural matching) was used to find the interaction between Vpr and A3G. Furthermore, the co-IP experiments proved this interaction. Vpr suppressed A3G through VprBP mediated proteosomal degradation. These results also suggest that there is a cross talk between different HIV-1 host ubiquitin complex systems as A3G is counteracted by both Vpr and Vif via proteosomal degradation (104).

RNA-associated early-stage antiviral factor (REAF) inhibits the replication of HIV1, HIV-2, and SIV (15). HIV-1 Vpr interacts with REAF to degrade REAF, and since REAF inhibits viral replication, therefore the degradation of REAF efficiently restores HIV-1 replication in primary macrophages (45). The infection of HeLa-CD4 cells and monocyte-derived macrophages (MDMs) by HIV-1 containing Vpr, decreases REAF levels in the nucleus within two hours of infection; however, when these cells are infected with HIV-1 that does not contain Vpr, the quantities of REAF in the nucleus increase as early as within 30 minutes. This indicates that in the absence of Vpr, HIV infection increases REAF levels and inhibits viral replication; however, in the presence of Vpr, REAF is degraded and as a result, viral replication is promoted (45).

Vpr has also been observed to degrade TET2 by VprBP-DDB1-CUL4-ROC1 E3 ligase (16). The degradation of TET2 due to Vpr increases HIV-1 replication, and significantly sustains interleukin-6 expression (IL-6). HIV-1 Vpr transfection into THP1 monocyte cells results in the reduction of TET2 protein levels; however, Vpr-deficient mutant HIV-1 does not reduce TET2 protein levels. Furthermore, Vpr degrades TET2 through Vpr binding protein (VprBP). Overexpression of TET2 inhibits HIV-1 replication, while knockout of TET2 significantly increases HIV-1 replication. HIV-1 Vpr infection of TET2 knockdown cells increases HIV-1 replication by approximately 4 to 5 fold (16).

Similarly, Vpr inhibit the nuclear translocation of IRF3 and NF-κB to antagonize several pathogen associated molecular patterns (PAMPS) (105). Vpr interact with karyopherins to inhibit IRF3 and NF-Κb nuclear translocation and thus promoting HIV replication in macrophages. This study thus demonstrates a model where Vpr inhibit innate immune activation by interacting karyopherins and therefore promote viral transmission (105).

## Vpx overcomes SAMHD1, STING, and HUSH restriction to promote HIV-2 replication

Sterile Alpha Motif and Histidine Aspartate domain containing protein 1 (SAMHD1) is an HIV-1 restriction factor in dendritic cells (DCs) and in non-dividing monocytes, resting CD4+ T cells and macrophages (106). SAMHD1 is highly expressed in DCs and macrophages where they decrease the cellular dNTP pool, and thereby impairs HIV-1 reverse transcription (19). When CD4+ T-cells are infected with HIV-1, HIV-1 RNA is reversed transcribed into DNA, which is a synthesis step that consumes cellular dNTPs. SAMHD1 hydrolyzes all four dNTPs into inorganic triphosphate and deoxynucleotides, thereby controlling the pool of cytosolic dNTPs. Similarly, in myeloid cells, SAMHD1 inhibits proviral DNA formation and HIV-1 replication (17, 19, 107). SAMHD1 also exhibits RNAase activity, targeting viral RNA for degradation before it is converted into DNA, and therefore restricts viral replication. However, the extent to which SAMHD1 may restrict HIV-1 replication through its RNAase activity remains speculative (108–111). Defective HIV-2virions, e.g., SIVsm, that infect sooty mangabey and SIVmac, that infect rhesus macaques) encode an accessory protein, Vpx, to counteract SAMHD1 activity (**Table 1**, **Figure 1**) (112). However, HIV-1 and simian immunodeficiency virus ancestor infecting chimpanzees (SIVcpz) do not encode Vpx, and therefore these viruses are exposed to the action of SAMHD1 (17). Vpx physically interacts with SAMHD1 at its C-terminal domain, thus recruiting the Cullin-4 E3 ubiquitin ligase complex, and destines SAMHD1 for poly-ubiquitination and proteasomal degradation (17–19).

The potential role of SAMHD1 in innate and adaptive immune responses have also been observed (113). SAMHD1 does contribute HIV-1 antigen presentation via MHC-1 molecules in monocyte-derived dendritic cells (DCs). SAMHD1 facilitates the inhibition of HIV-1 replication while Vpx-mediated depletion of SAMHD1 increases HIV-1 antigen presentation by DCs, leading to the activation of HIV-1 specific cytotoxic T-lymphocyte (CTL) responses, and the subsequent killing of DCs (113).

The depletion of SAMHD1 induces IFN-1 production, which is a HIV/SIV inhibitor (114). In the absence of SAMHD1, cyclic GMP-AMP synthase (cGAS) and stimulator of IFN genes (STING) regulate the DNA-sensing pathway, leading to the activation of IFN production. It has been found that interaction between Vpx and STING is required for STING inhibition, and interestingly, Vpx interacts with the domain of STING that is required for NF-κB activation. Therefore, Vpx inhibits NF-κB activation (which is mediated by cGAS-STING) to promote viral infection (46).

The human silencing hub (HUSH) complex is a complex of transcription activation suppressor (TASOR), M-phase phosphoprotein 8 (MPP8), and periphilin, and is involved in the silencing of the transcriptional system (115). HUSH complex is a host restriction factor; however, HUSH is suppressed by HIV-2 Vpx via the interaction of HIV-2 Vpx with HUSH. HIV-2 Vpx recruits DCAF1 ubiquitin ligase adopter to degrade HUSH (47). Interestingly, HUSH is not antagonized by HIV-1 Vpx, and is only antagonized by SIVs and HIV-2 (47).

## Conclusions and future perspectives

Researchers have, in recent times, focused on the host restriction factors that serve as natural host defenses against HIV-1. In the present review article, we have summarized the host restriction factors that are known to be targeted by HIV proteins and thus to facilitate HIV pathogenesis. We have ascertained the following: Nef inhibits the activity of Ser5, PSGL1, and IFITIMs; Vif antagonizes APOBEC3G; Vpu antagonizes tetherin, PSGL1 and GBP5; Env restrict tetherin and Ser5; Vpr counteracts REAF and TET2, whereas Vpx of HIV-2 overcomes SAMHD1, STING, and HUSH complex restriction to promote HIV replication. Since Nef antagonizes three individual host proteins, i.e., Ser5, PSGL1, and IFITIMs, and Vpx also antagonizes three host restriction factors, i.e., SAMHD1, STING, and the HUSH complex, we believe that these proteins may well be categorized as potential candidates for future HIV therapeutics.

There are some limitations in the previous literature as well. Previous studies have not focused on the levels of host protein antagonism by HIV proteins. Moreover, there are HIV proteins which counteract more than one host proteins, therefore, none of the previous studies focused on that particular HIV protein that antagonized more than one host protein. More robust evidences are lacking that support a direct interaction between Env glycoprotein and Ser5.

In the future, host restriction factors might be of keen interest to researchers, and host restriction factors and inhibitors which specifically target their respective HIV proteins at different stages of the HIV life cycle may well be able to be utilized to inhibit HIV-1 pathogenicity, replication, and infectivity. Furthermore, certain host restriction factors target HIV to antagonize HIV infection; however, the underlying mechanisms whereby HIV co-opts and counteracts those restriction factors remain elusive, and remain to be determined. HIV-1 counteracts PSGL-1 at an early time point during reverse transcription; however, the fundamental mechanisms underlying this process remain unclear. Similarly, the process whereby PSGL-1 inactivates virion infectivity remains elusive. In the future, focus should be diverted to those HIV proteins which target a higher number of host restriction factors, as this may be of interest for HIV therapeutic purposes. The polymorphism that occurs with Nef impairs its ability to internalize Ser5; therefore, the varying mutations of Nef protein may possibly contribute to variances in viral pathogenesis. Future focused studies will enhance our understanding of the Nef-Ser5 interface at molecular levels, and this may well represent a potential investigational realm for the discovery of novel antiretroviral therapeutics. HIV-1 restricts Ser5 via two means, i.e., Nef and Env; therefore, exploration of the question as to why HIV-1 has evolved dual means to antagonize Ser5 will also be an interesting area of study in the future. In the present review article, we have shown that the HIV-1 protein, Nef, targets three host restriction factors to facilitate its pathogenesis, i.e., Ser5, PSGL1, and IFITM. We therefore believe that Nef may be a potentially productive option to be targeted for therapeutic purposes in future studies.
